# The Responsibility for Diseased Meat and Milk

**Published:** 1907-08-10

**Authors:** 


### The Hospital

A Journal of

The Medical Sciences and Hospital Administration.

New Series. No. 20, Vol. I. [No. 1092, Vol. XLII.] SATURDAY, AUGUST 10, 1907.

### The Responsibility for Diseased Meat and Milk.

In an article published in the current issue of the *Nineteenth Century*, Dr. A. Mearns Fraser, Medical Officer for Health for Portsmouth, endeavours to arouse public opinion to the dangers which exist from the distribution of diseased meat and milk; and by an analysis of existing facts he also tries to show where the responsibility for such distribution rests. There is no attempt to work up a sensation or to exploit some particular suggestion or fad. On the contrary, the article is mainly based on personal experiences and on official documents, and it may therefore be accepted as a statement of actually existing conditions. Hence, whether one agrees or disagrees with Dr. Fraser's conclusions, it is necessary to recognise the *bona fides* of his communication, and to listen to his arguments with all respect. Much of what he says is by no means new, but as it is certain that no effective measures to prevent the dangers he describes will ever be taken unless under the pressure of public opinion, repetition of the essential facts is advantageous and even necessary. The adoption of the methods by means of which preventive medicine can continue to enlarge the sphere of its successful operations depends in the last resort upon popular conviction and enlightenment. Therefore such methods must be discussed and urged in journals which reach the public ear. Hence, the influence exerted by diseased meat and milk in the production of disease, and the arrangements necessary to neutralise this influence, cannot be too frequently presented to the attention of the community.

Confining his attention for the most part to tuberculosis, Dr. Fraser asserts, in the first place, that tuberculous milk and meat are both widely supplied to the public; and he has taken steps to verify this as part of his official duty. In Portsmouth, as in many other towns, there is no public abattoir, and as slaughtering may take place at any hour in the various private establishments, it is impossible to secure the efficient and confident examination of all the animals killed for food. To meet this position it was determined to send an inspector to the various cattle markets in the neighbourhood, and thus to gain information of any animal having a suspicious appearance that might be sold to a local dealer. The animal was then followed, and as soon as it came within the jurisdiction of the municipality it was examined and, if necessary, destroyed. In this ingenious fashion the inhabitants of Portsmouth were protected from a supply of tuberculous meat. But even this measure cannot prevail against the sale of infected milk, and Dr. Fraser expressly asserts that numbers of the animals recognised by his inspectors to be tuberculous had, long prior to the date on which they were offered for sale, been employed for the supply of milk.

The question inevitably arises, How may this position be met? The radical defect of existing legislation, it is claimed, is that its administration is entrusted to those who either are incompetent or are disinclined conscientiously to enforce its requirements. The animals are not inspected on the farms, but are brought for sale to the cattle market of some neighbouring town. Here, of course, an opportunity for examination exists, and it is within the power of the local authority to seize any animal that appears diseased, unsound, or unwholesome. But the sanitary authorities in country towns are composed largely of local tradesmen and farmers, who are financially interested in the rearing and sale of cattle. Not unnaturally, therefore, they are not actively critical of the quality of the animals exposed for sale within their jurisdiction, and are disposed to leave the detection of disease to the inspectors of the towns where the animals are slaughtered. And this, as has been already pointed out, cannot be relied on in places where there are numerous private slaughter-houses and not a public abattoir. It may be said that it is the duty of the medical officer in the town of sale to detect and seize diseased cattle. Granted; but a medical officer who endeavours to enforce health requirements which affect the pockets of the district councillors has not infrequently found difficulty in obtaining the votes necessary to secure his next annual reappointment. Similarly, in connection with the milk supply, the consumer has no efficient check on the producer, whose interest it is to obtain the largest quantity of milk at the least possible expense. Here, too, the sanitary authorities, in the shape of the district councils, include farmers and others pecuniarily interested in the milk trade. Hence it is hardly a matter for wonder that these bodies have never shown an inclination to put into effective operation even the general provisions of the Public Health Acts, far less to apply the regulations relating to the inspection and conduct of dairy farms. However excellent may be existing legislation in these directions, it remains a dead letter so long as its administration is in the hands of those whose immediate interests are more or less antagonistic to its claims. The remedy seems to lie in a transference of the administration to a larger unit of local administration, or perhaps to the Local Government Board. Short of this, it is essential that the local sanitary officers, and, in particular, medical officers of health, shall not be deprived of their positions on the mere fiat of the local authorities. Until this reform is adopted, impartiality and efficiency on the part of these officers are impossible. Both of these proposals need popular force to ensure their adoption, and Dr. Fraser has done well to announce their necessity to the public.

